# Physiological effects of high-flow oxygen in tracheostomized patients

**DOI:** 10.1186/s13613-019-0591-y

**Published:** 2019-10-07

**Authors:** Daniele Natalini, Domenico L. Grieco, Maria Teresa Santantonio, Lucrezia Mincione, Flavia Toni, Gian Marco Anzellotti, Davide Eleuteri, Pierluigi Di Giannatale, Massimo Antonelli, Salvatore Maurizio Maggiore

**Affiliations:** 1 Department of Anesthesiology and Intensive Care, Catholic University of the Sacred Heart, Fondazione ‘Policlinico Universitario A. Gemelli’ IRCCS, Rome, Italy; 2 Department of Medical, Oral and Biotechnological Sciences, School of Medicine and Health Sciences, Section of Anesthesia Analgesia, Perioperative and Intensive Care, SS. Annunziata Hospital, Gabriele d’Annunzio University of Chieti-Pescara, Via dei Vestini, 66100 Chieti, Italy

**Keywords:** Oxygen inhalation therapy, Tracheostomy, Respiratory insufficiency, Mechanical ventilator weaning, Positive end-expiratory pressure

## Abstract

**Background:**

High-flow oxygen therapy via nasal cannula (HFOT_NASAL_) increases airway pressure, ameliorates oxygenation and reduces work of breathing. High-flow oxygen can be delivered through tracheostomy (HFOT_TRACHEAL_), but its physiological effects have not been systematically described. We conducted a cross-over study to elucidate the effects of increasing flow rates of HFOT_TRACHEAL_ on gas exchange, respiratory rate and endotracheal pressure and to compare lower airway pressure produced by HFOT_NASAL_ and HFOT_TRACHEAL._

**Methods:**

Twenty-six tracheostomized patients underwent standard oxygen therapy through a conventional heat and moisture exchanger, and then HFOT_TRACHEAL_ through a heated humidifier, with gas flow set at 10, 30 and 50 L/min. Each step lasted 30 min; gas flow sequence during HFOT_TRACHEAL_ was randomized. In five patients, measurements were repeated during HFOT_TRACHEAL_ before tracheostomy decannulation and immediately after during HFOT_NASAL_. In each step, arterial blood gases, respiratory rate, and tracheal pressure were measured.

**Results:**

During HFOT_TRACHEAL_, PaO_2_/FiO_2_ ratio and tracheal expiratory pressure slightly increased proportionally to gas flow. The mean [95% confidence interval] expiratory pressure raise induced by 10-L/min increase in flow was 0.2 [0.1–0.2] cmH_2_O (*ρ* = 0.77, *p* < 0.001). Compared to standard oxygen, HFOT_TRACHEAL_ limited the negative inspiratory swing in tracheal pressure; at 50 L/min, but not with other settings, HFOT_TRACHEAL_ increased mean tracheal expiratory pressure by (mean difference [95% CI]) 0.4 [0.3–0.6] cmH_2_O, peak tracheal expiratory pressure by 0.4 [0.2–0.6] cmH_2_O, improved PaO_2_/FiO_2_ ratio by 40 [8–71] mmHg, and reduced respiratory rate by 1.9 [0.3–3.6] breaths/min without PaCO_2_ changes. As compared to HFOT_TRACHEAL_, HFOT_NASAL_ produced higher tracheal mean and peak expiratory pressure (at 50 L/min, mean difference [95% CI]: 3 [1–5] cmH_2_O and 4 [1–7] cmH_2_O, respectively).

**Conclusions:**

As compared to standard oxygen, 50 L/min of HFOT_TRACHEAL_ are needed to improve oxygenation, reduce respiratory rate and provide small degree of positive airway expiratory pressure, which, however, is significantly lower than the one produced by HFOT_NASAL_.

## Background

Nasal high-flow oxygen therapy (HFOT_NASAL_) has been proposed to treat acute hypoxemic respiratory failure [1–4], to facilitate weaning from mechanical ventilation [5–8] and to prevent hypoxemia during endotracheal intubation [9, 10].

With HFOT_NASAL_, up to 60 L/min of heated and humidified air/oxygen mixture are continuously delivered to the patient through specifically designed nasal prongs [11]. Unlike standard oxygen, high flows limit dilution of inhaled gas mixture, thus enabling more accurate delivery of the set fraction of inspired oxygen (FiO_2_) [12]. HFOT_NASAL_ increases end-expiratory lung volume due to the generation of flow-dependent airway positive pressure, with highest values reached at end-expiration with closed mouth [13–15]. The continuous high flow washes CO_2_ out from upper airways, reducing anatomical dead space and work of breathing [16]. Active heating/humidification and the comfortable interface improve comfort related to airway dryness and optimize device tolerability [16–18].

High-flow oxygen can be delivered also through tracheostomy (HFOT_TRACHEAL_), but its mechanism of action and physiological effects appear different and have not been thoroughly elucidated [19, 20]. We conducted a randomized cross-over study to assess the effects of HFOT_TRACHEAL_ administered at different gas flow rates on gas exchange, tracheal pressure, and respiratory rate, and to establish whether the increase in airway pressure generated by high-flow oxygen is different when administered by nasal cannula or tracheostomy.

## Methods

The present study was carried out in the general intensive care unit (ICU) of a tertiary-care university hospital in Rome between September 2016 and September 2017, after a preliminary study conducted on a previous cohort of patients to assess the feasibility of tracheal pressure measurement in critically ill patients [21]. The study protocol was approved by the local institutional review board; written informed consent was obtained by all patients or next of kin, according to the ethics committee recommendations.

### Patients

We studied critically ill tracheostomized patients with no hemodynamic instability who had been weaned from mechanical ventilation, had been spontaneously breathing with no ventilatory support for at least 24 h and were receiving tracheal oxygen according to the prescription of the attending physician. All enrolled patients had received single-dilator percutaneous tracheostomy with PercuTwist^®^ technique (Rüsch, Kernen, Germany): the procedure was performed by an intensivist under bronchoscopy, which confirmed that the puncture was taking place between the first and second, or second and third, tracheal rings [22, 23]. Non-inclusion criteria were age < 18 years, pregnancy, recent tracheal, esophageal, neck or thoracic surgery, presence of pneumothorax/chest drainage. For safety reasons, patients with partial pressure of arterial oxygen to nominal FiO_2_ ratio (PaO_2_/FiO_2_) below 100 mmHg and/or respiratory rate > 45 breaths per minute during standard oxygen were not enrolled.

### Procedures

After study inclusion, each patient received for 30 min standard oxygen through tracheostomy with a heat and moisture exchanger (Tracheolife II HME, Mallinckrodt, United Kingdom), with oxygen flow set by the attending physician (*standard oxygen* step, maximal O_2_ flow 8 L/min).

Patients subsequently underwent high-flow oxygen: gas flow was provided by the dedicated module of an ICU ventilator (EvitaXL or EvitaInfinity, Drager, Lubeck, Germany), inspired gas was actively conditioned by heated humidifier set at 37 °C (HH MR850, Fisher & Paykel Healthcare, New-Zealand, absolute humidity provided 44 mgH_2_O/L) and delivered through the specifically designed interface (Optiflow™ Tracheostomy interface OPT870, Fisher & Paykel Healthcare, New-Zealand). Three oxygen flow rates with the HFOT_TRACHEAL_ device were tested in random order, for 30 min each: 10 L/min, 30 L/min, and 50 L/min. No wash-out period was applied between these interventions. Although 10 L/min cannot be considered as ‘high-flow therapy’, this step allowed (A) to better characterize the effects of increasing flow rate with the same device on analyzed endpoints, and (B) to compare standard oxygenation device (closed system through a heat a moisture exchanger) and HFOT_TRACHEAL_ (open system) at similar gas flow rate, highlighting the difference between these techniques. The randomization sequence was provided by S.A.S. random allocation software. FiO_2_ was set to obtain a SpO_2_ between 92 and 98% (88–92% in patients with PaCO_2_ ≥ 45 mmHg during standard oxygen). Changes in the FiO_2_ over the course of the study were discouraged and allowed only whether clinically unavoidable.

### Measurements

At the end of each step, hemodynamic parameters, arterial blood gases and SpO_2_ were recorded. To estimate PaO_2_/FiO_2_ during standard oxygen, delivered FiO_2_ was calculated using a previously described formula [24]:

$${\text{FiO}}_{2} = \left( {{\text{oxygen}}\;{\text{flow rate}}\; {\text{in}}\;{\text{liters}}\;{\text{per}}\;{\text{minute}} *\;0.03} \right) + 0.21.$$

At study entry, a sterile, disposable 18-gauge catheter (15/25-cm length according to patient’s height; 1-mm diameter; BD, CareFusion corporation, San Diego, CA, USA) connected to a differential pressure transducer was inserted in the trachea (2 cm away from carina, with the distance between tracheal stoma and carina measured on the chest X-ray) and secured to the skin with an adhesive tape. At the end of each study step, endotracheal pressure was recorded continuously for 3 min by a dedicated software at a sample rate of 200 Hz (Kleis-Tek, ICU lab, Bari, Italy). Pressure signals were offline-reviewed to assess respiratory rate and compute mean expiratory pressure (between the end of inspiration and the beginning of the following inspiration), peak expiratory and inspiratory pressure (maximal and minimal pressure achieved over the whole respiratory cycle, respectively). All these parameters were measured for all breaths in the 3-min recording and values were averaged for each study step.

In a subgroup of five patients who underwent tracheostomy decannulation after study inclusion and during the ICU stay, the experimental protocol was repeated on the day of decannulation, both during HFOT_TRACHEAL_ and during HFOT_NASAL_ after decannulation. Briefly, when the tracheal cannula was removed, the catheter for tracheal pressure measurement was hold in situ and the stoma was covered with gauze and adherent sealing tape (percutaneous tracheostomy maintains subcutaneous tissue integrity and elasticity) [25]. After medication, absence of leaks through the stoma was assessed by hand while the patient spontaneously vocalized and coughed. This approach was clinically useful for assessing patient’s tolerance to mouth/nose breathing and represented a unique opportunity to evaluate lower airway pressure during HFOT_NASAL_. In these 5 patients, HFOT_TRACHEAL_ and HFOT_NASAL_ with three flow settings (10, 30 and 50 L/min) were applied for 20-min periods in sequential order, just before and immediately after tracheostomy decannulation. No wash-out period was applied between the interventions. Heated humidifier settings were kept unchanged. Towards the end of each period, tracheal pressure tracings were recorded and were offline-analyzed to compute mean and peak expiratory pressure, as previously described.

### End-points

Primary endpoint was to compare ratio of arterial oxygen partial pressure to nominal FiO_2_ (PaO_2_/FiO_2_) in the different study steps. Main secondary endpoints were to analyze the effects of the tested settings on respiratory rate, endotracheal pressure and PaCO_2_. Furthermore, we aimed at establishing whether tracheal pressure is different when high-flow oxygen is delivered through tracheostomy or nasal cannula, at similar flow rates.

### Statistical analysis

Descriptive data are expressed as number and percentage and continuous data as median [interquartile range]. Because of the limited sample, adopting a conservative approach, all data were analyzed with non-parametric tests. Paired comparisons between the study steps were performed with the Wilcoxon sum of ranks test and mean differences [95% confidence interval] are displayed for most significant results. Correlation was assessed with Spearman’s rank-order correlation: *ρ* and the *p* value are reported. Analysis on the mean expiratory pressure rise induced by increasing gas flow was performed with linear regression: the slope and the *p* value of the relationship are reported. Inter-individual variability was rated with the coefficient of variation, computed as the ratio of standard deviation to mean of the measurements [26]. Results with two-tail *p* ≤ 0.05 were considered significant. Statistical analysis was performed with SPSS 20.0 (IBM SPSS Statistics for Windows, Version 20.0. Armonk, NY, USA).

### Sample size

Clinical data on the effects of HFOT_TRACHEAL_ are limited to a single exploratory study [20]: this hampered any estimation of the adequate sample needed to provide sufficient statistical power to the study. Because previous investigations with similar design demonstrate that 15–20 patients studied in a cross-over fashion represent an adequate sample to draw conclusions on similar physiological endpoints [13, 15, 18, 20, 27], adopting a conservative approach, we planned to enroll 25 patients.

## Results

Twenty-six patients were enrolled and analyzed. Demographics and most relevant clinical characteristics are reported in Table 1. In the standard oxygen step, median oxygen flow was 4 [3, 4] L/min and median estimated FiO_2_ was 0.33 [0.33–0.37]. No patient experienced changes in heart rate or arterial blood pressure over the course of the study. The sequence of HFOT_TRACHEAL_ interventions did not affect PaO_2_/FiO_2_ (*ρ* = 0.05, *p* = 0.69) nor respiratory rate (*ρ* = 0.002, *p* = 0.99).

**Table 1:** Baseline characteristics of enrolled patients

No. of patients	26
Age, years	57 [48–71]
Female sex, no. (%)	4 (15)
Height, cm	175 [168–180]
Body weight, kg	75 [70–85]
Body mass index, kg/m^2^	25 [24–28]
SAPS II	46 [41–60]
Patients with history of COPD, no. (%)	5 (19)
ICU admission, no. (%)	
Medical	12 (46)
Surgical	7 (27)
Trauma	7 (27)
Cause of prolonged need for mechanical ventilation, no (%)	
Respiratory failure	8 (31)
Traumatic brain injury	7 (27)
Non-traumatic brain injury	11 (42)
Length of mechanical ventilation before enrollment, days	11 [8–13]
Glasgow coma scale at enrollment	10 [6–15]
PaO_2_/FiO_2_ during standard oxygen, mmHg^a^	238 [197–311]
Tracheal cannula inner diameter, mm	9 [8.5–10]
Tracheal cannula external diameter, mm	12.3 [12.3–12.3]
Length of ICU stay, days	20 [14–26]
In-ICU mortality, no. (%)	3 (12)

Results are displayed as medians [interquartile range], if not otherwise specified

*SAPSII* simplified acute physiology score 2 at ICU admission, *COPD* chronic obstructive pulmonary disease, *ICU* intensive care unit

^a^Measured during the standard oxygen step of the experiment

### Gas exchange and respiratory rate

**Fig. 1:**
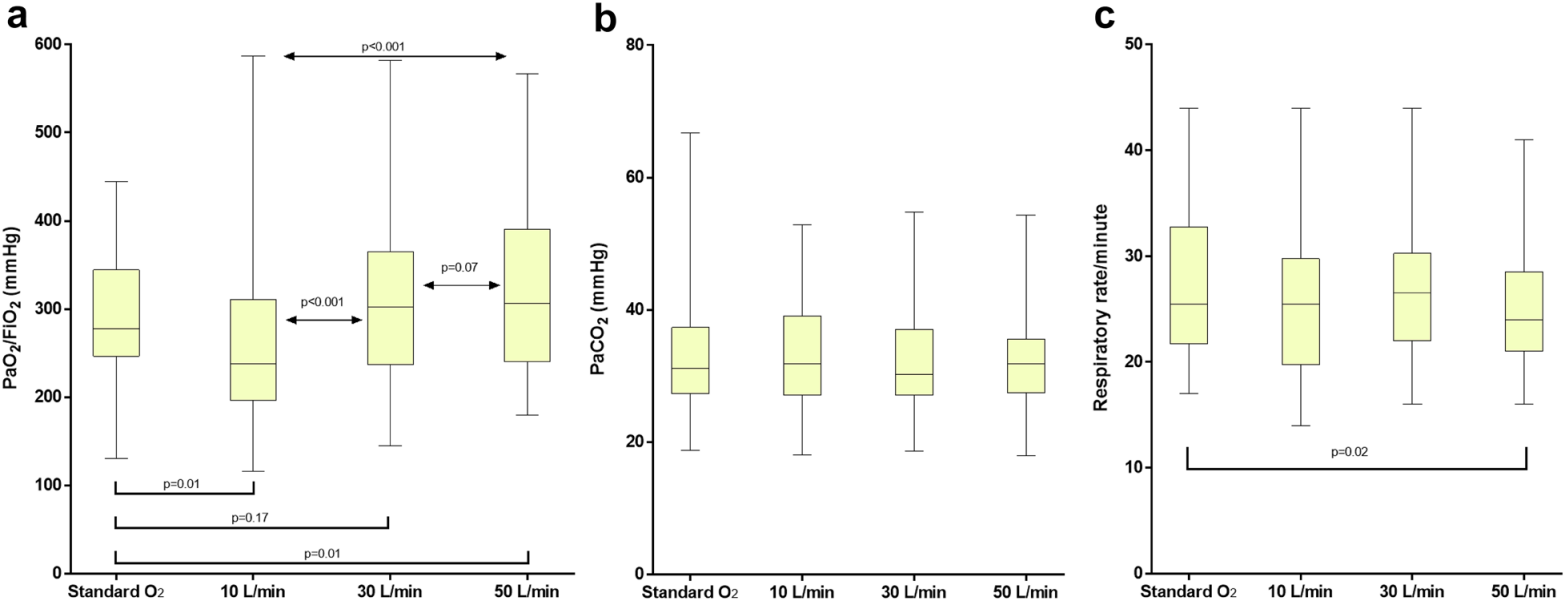
PaO_2_/FiO_2_ (**a**), PaCO_2_ (**b**) and respiratory rate (**c**) in the four study steps. Results are displayed as median, interquartile range, maximum and minimum. With HFOT_TRACHEAL_ device, PaO_2_/FiO_2_ increases proportionally to gas flow, especially between 10 and 30 L/min. As compared to standard oxygen, 50 L/min, but not 30 L/min nor 10 L/min, ameliorate oxygenation and reduce respiratory rate in isocapnic conditions

These results are displayed in Fig. 1.

During HFOT_TRACHEAL_, increasing flow rates yielded improvement in oxygenation, markedly between 10 and 30 L/min (*p* < 0.001) and mildly between 30 and 50 L/min (*p* = 0.07).

As compared to standard oxygen, HFOT_TRACHEAL_ 50 L/min, but not 30 nor 10 L/min, increased PaO_2_/FiO_2_ ratio: median [Interquartile range] 307 [241–390] mmHg vs. 277 [247–344] mmHg, *p* = 0.01; mean difference [95% CI] 40 [8–71] mmHg) (Fig. 1a).

When compared to standard oxygen, HFOT_TRACHEAL_ 50 L/min led to a slight reduction in respiratory rate (24 [21–29] breaths/min vs. 26 [22–33] breaths/min, *p* = 0.02), without changes in PaCO_2_ (32 [26–36] mmHg vs. 31 [27–37] mmHg, *p* = 0.43) (Fig. 1b, c). The mean reduction [95% CI] in respiratory rate yielded by HFOT_TRACHEAL_ 50 L/min was 1.9 [0.3–3.6] breaths/min and was proportional to respiratory rate during standard oxygen (i.e., greater in patients with higher respiratory rate, *ρ* = 0.43 *p* = 0.03). No differences in PaCO_2_ were detected between the studied conditions (Fig. 1b, c).

#### Tracheal pressure

**Fig. 2:**
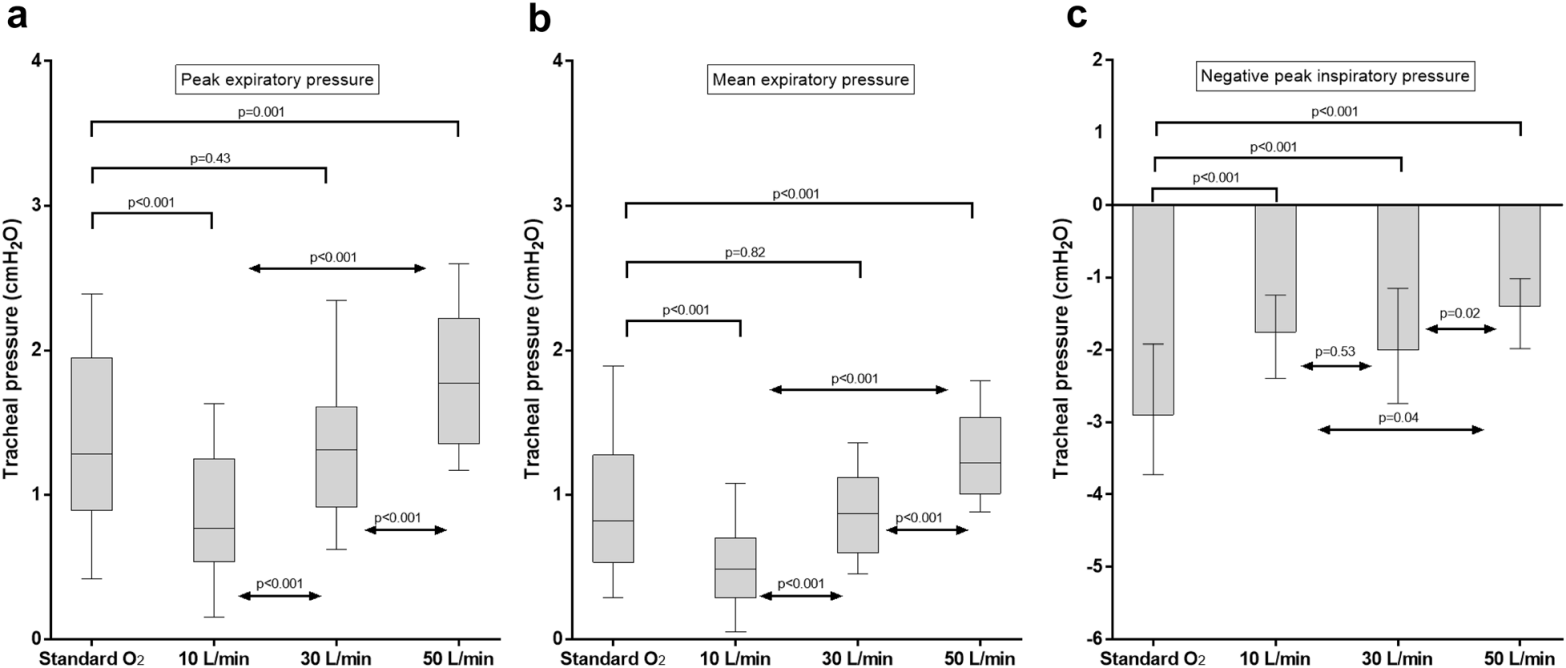
Peak (**a**), mean expiratory pressure (**b**) and negative peak of inspiratory pressure. Results are displayed as median, interquartile range, maximum and minimum. During HFOT_TRACHEAL_, tracheal expiratory pressure increases proportionally to the gas flow. All HFOT_TRACHEAL_ settings limit the negative inspiratory pressure, especially as flow is set at 50 L/min, likely due to the capability of the high gas flow in an open system to match patient’s peak inspiratory flow. As compared to standard oxygen, 50 L/min, but not 30 L/min nor 10 L/min, increase tracheal peak and mean tracheal expiratory pressure

These results are displayed in Fig. 2.

In the three HFOT_TRACHEAL_ steps, mean and peak expiratory pressures were proportional to the delivered gas flow (*p* < 0.001 for all comparisons). The mean [95% CI] expiratory pressure rise induced by 10-L/min increase in flow was 0.2 [0.1–0.2] cmH_2_O (*ρ* = 0.77, *p* < 0.001). As compared to standard oxygen, 50 L/min, but not other HFOT_TRACHEAL_ settings, led to an increase in peak and mean expiratory pressures: peak pressure 1.8 [1.4–2.2] cmH_2_O vs. 1.3 [0.9–2] cmH_2_O, *p* = 0.001; mean pressure 1.2 [1–1.5] cmH_2_O vs. 0.8 [0.5–1.3] cmH_2_O, *p* < 0.001 (Fig. 2a, b). Mean differences [95% CI] in peak and mean expiratory pressure between HFOT_TRACHEAL_ 50 L/min and standard oxygen were 0.4 [0.2–0.6] cmH_2_O and 0.4 [0.3–0.6] cmH_2_O, respectively. Both peak and mean expiratory pressures were lower at HFOT_TRACHEAL_ 10 L/min than during standard oxygen (both *p* < 0.001).

All HFOT_TRACHEAL_ settings yielded less negative tracheal peak inspiratory pressure, as compared to standard oxygen (*p* < 0.001 for all the comparisons): this effect was magnified at 50 L/min (Fig. 2c).

### Comparison with HFOT_NASAL_

**Fig. 3:**
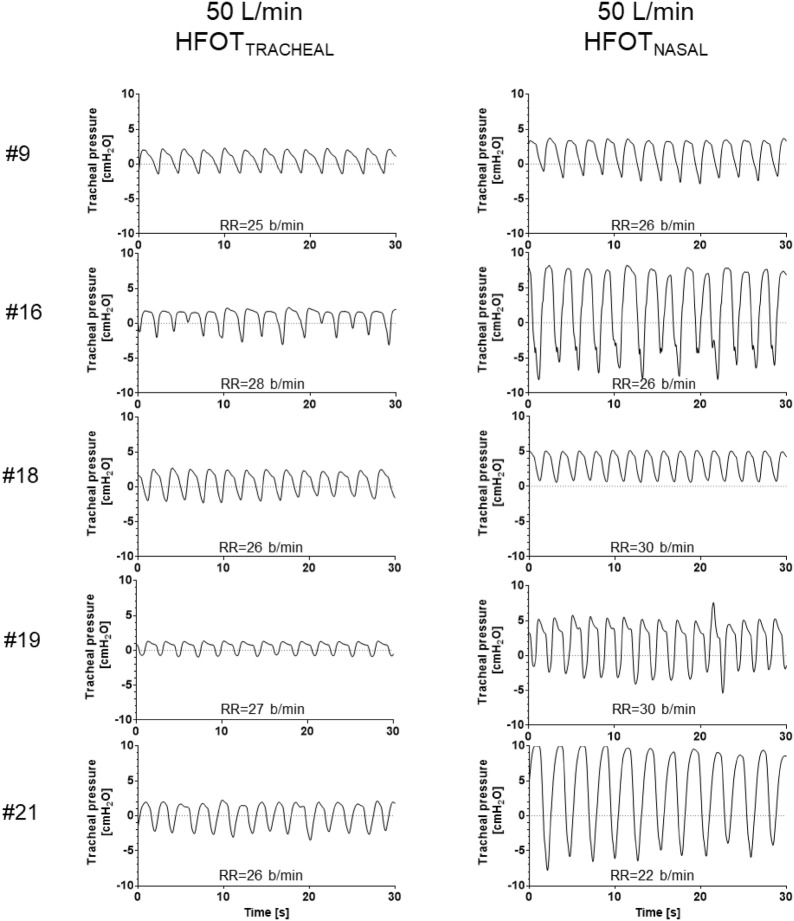
Thirty-second recordings of tracheal pressure tracings during HFOT_TRACHEAL_ and HFOT_NASAL_ in 5 patients who underwent tracheostomy decannulation over the course of ICU stay. In both conditions gas flow was set at 50 L/min. Average respiratory rate for the 30-s recording is reported for all conditions. During HFOT_NASAL_ lower airway pressure during expiration is higher and more inter-individually variable than HFOT_TRACHEAL_, despite a non-dissimilar respiratory rate, which was calculated on the same 30-s recording. This suggests that the HFOT_NASAL_-induced increase in expiratory pressure depends not only on gas flow, but also on patient’s expiratory pattern and, likely, on individual respiratory system mechanical properties. Please note that, under this condition, tracheal pressure was not constant over the course of the respiratory cycle and became negative during inspiration in 4 patients, which is different from what previously reported for pharyngeal pressure [14]

**Fig. 4:**
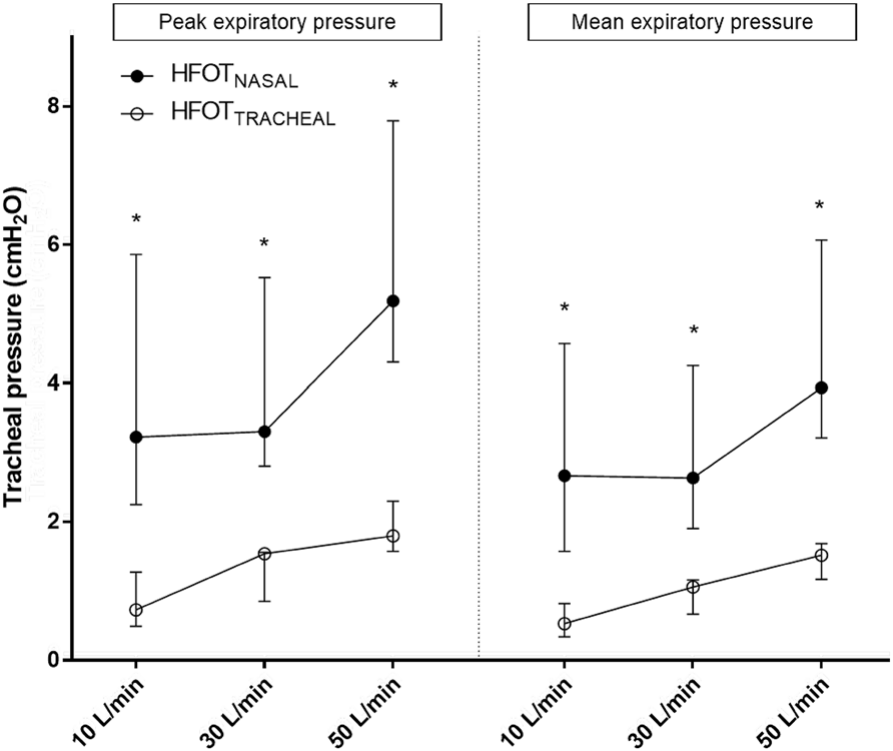
Peak and mean expiratory pressure during HFOT_TRACHEAL_ and HFOT_NASAL_ and different gas flows delivered. Results are displayed as median and interquartile range; *indicates *p* ≤ 0.05 for HFOT_TRACHEAL_ vs. HFOT_NASAL_ comparisons

Five patients underwent tracheostomy decannulation within their stay in ICU, and received HFOT_TRACHEAL_ and HFOT_NASAL_ before and after the procedure. Samples of tracheal pressure tracings are displayed in Fig. 3. Inter-individual variability in peak and mean expiratory pressure at 50 L/min was greater during HFOT_NASAL_ (both 35%) than during HFOT_TRACHEAL_ (21 and 20%, respectively). Inspiratory pressure during HFOT_NASAL_ 50 L/min fell below 0 during inspiration in 4/5 patients. With all the tested flow settings, peak and mean expiratory tracheal pressures during HFOT_NASAL_ were significantly higher than during HFOT_TRACHEAL_ (Fig. 4; *p* = 0.05 for all comparisons). In particular, with flow set at 50 L/min: median peak expiratory pressure was 5.1 [4.2–7.7] cmH_2_O during HFOT_NASAL_ vs. 1.8 [1.6–2.3] cmH_2_O during HFOT_TRACHEAL_ (*p* = 0.05); mean expiratory pressure was 3.9 [3.1–6] cmH_2_O during HFOT_NASAL_ vs. 1.5 [1.2–1.7] cmH_2_O during HFOT_TRACHEAL_ (*p* = 0.05). The mean difference [95% CI] in tracheal peak and mean expiratory pressure between HFOT_NASAL_ and HFOT_TRACHEAL_ was 4 [1–7] cmH_2_O and 3 [1–5] cmH_2_O, respectively.

## Discussion

In the present cross-over study, we show that, as compared to standard oxygen, HFOT_TRACHEAL_ mitigates the negative swing in airway pressure during inspiration, and, when flow is set at 50 L/min, ameliorates oxygenation and slightly reduces respiratory rate. With similar flow rates, tracheal expiratory pressure is significantly lower with HFOT_TRACHEAL_ than with HFOT_NASAL_, suggesting that the physiologic effects of HFOT_TRACHEAL_ are milder than HFOT_NASAL_. A gas flow of 50 L/min should be set with the tracheal interface to slightly improve oxygenation and reduce respiratory rate.

Several studies addressed the effects of HFOT_NASAL_ in a variety of clinical *scenarii* [1]. Although high-flow oxygen can be delivered through tracheostomy, few data elucidate its mechanisms of action, which can be different from HFOT_NASAL_ [20].

### Oxygenation

During HFOT_TRACHEAL_, PaO_2_/FiO_2_ ratio increases proportionally to gas flow. However, when compared to standard oxygen via heat and moisture exchangers, only 50 L/min generate improvement in PaO_2_/FiO_2_ ratio. These data are partially consistent with what has been reported for HFOT_NASAL_ [18] and may be explained by the following mechanisms:

1. Increasing flow rate up to 50 L/min can limit air dilution of inhaled gas mixture, enabling more accurate delivery of set FiO_2_. This can be demonstrated by the reduction of the inspiratory airway pressure swing during HFOT_TRACHEAL_.
2. Increasing flow rate yields a concomitant increase in peak and mean expiratory pressure. Although the increase in tracheal pressure generated by HFOT_TRACHEAL_ is lower than the one reported during HFOT_NASAL_ [11, 14, 15, 28], this rise in expiratory pressure may still contribute to increase end-expiratory lung volume, reduce shunt fraction, optimize lung mechanics and improve oxygenation [11, 13, 18, 29].

One previous report showed that, when compared to T-Piece with a Venturi generator in tracheostomized patients, airway pressure and SpO_2_/FiO_2_ slightly increase during 50 L/min HFOT_TRACHEAL_ [20]. However, because of the entrainment effect, Venturi systems can provide flows up to 30–50 L/min and cannot be considered standard oxygen devices [30]. Standard oxygen through heat and moisture exchangers represents a widely used alternative for oxygen therapy in tracheostomized patients.

We have shown that standard oxygen through heat and moisture exchangers produces positive expiratory pressure, which is comparable to the one obtained with 30 L/min of high-flow oxygen through an open system. In fact, oxygenation between these two settings was similar. For the same gas flow (≈ 10 L/min), oxygenation and tracheal expiratory pressure were higher with the standard oxygenation (closed system) than with the HFOT_TRACHEAL_ device (open system). This suggests that the oxygenation changes are dependent on the amount of tracheal expiratory pressure. However, mechanisms of airway pressure generation may be different between the two devices: with standard oxygen, the increase in pressure depends on the expiratory resistance produced by the heat and moisture exchanger; while, during HFOT_TRACHEAL_, positive expiratory pressure is produced by patient’s expiration against the delivered gas flow in an open system and airway pressure is more stable over the respiratory cycle (i.e., less negative during inspiration). In this context, avoidance of excessive negative inspiratory swings in airway (and pleural) pressure is important to mitigate the risk of negative pressure pulmonary edema, whose occurrence induces lung damage and worsens oxygenation [31].

#### CO_2_ clearance

HFOT_NASAL_ lowers inspiratory resistance and enhances anatomical dead space clearance with CO_2_ washout [32, 33], finally reducing work of breathing [11, 13, 27, 34]. Our study shows that 50 L/min HFOT_TRACHEAL_ lowers respiratory rate without changes in PaCO_2_, as compared to standard oxygen. A reduction in respiratory rate has been reported during HFOT_NASAL_ [5, 35] and has been linked to anatomical dead space clearance, increased tidal volume, diminished resistive work of breathing and, in chronic obstructive pulmonary disease patients, increased positive expiratory pressure [13, 33, 36].

Work of breathing reduction by HFOT_NASAL_ is obtained at 30 L/min and is minimally enhanced by further increases in gas flow [18]: differently, 50 L/min of HFOT_TRACHEAL_ are needed to generate effects on respiratory rate. It is, therefore, reasonable to hypothesize that, in tracheostomized patients:

1. lower anatomical dead space and inspiratory resistance reduce the size effect of the intervention, that consequently requires higher flows to generate a significant effect;
2. inspired and expired flows are forcedly unidirectional, thus clearing anatomical dead space and improving breathing efficiency [37]: this contributes to CO_2_ washout independently from the device used for oxygen therapy, thereby mitigating the effect of HFOT_TRACHEAL_.

Our results are consistent with recent data indicating that HFOT_TRACHEAL_ minimally affects neuro-ventilatory coupling, work of breathing and gas exchange after weaning from mechanical ventilation [19].

### Differences with HFOT_NASAL_

Our comparison of HFOT_TRACHEAL_ and HFOT_NASAL_ in the same patients represented a unique opportunity to highlight the contribution of upper airway resistance to positive-pressure generation during HFOT_NASAL_. In fact, to our knowledge, no other data clarify the behavior of lower airway pressure during this treatment. The average expiratory pressure reported in our study is similar to what has been reported for pharyngeal pressure [11, 15, 28]. However, tracheal pressure during HFOT_NASAL_ was not constant over the respiratory cycle and became negative during inspiration in 4 of the 5 studied patients, which is different from what has been reported on upper airway pressure [14]. Our results indicate that expiratory pressure in lower airways is higher and more inter-individually variable when high flows are delivered through nasal cannula than through tracheostomy. This suggests that the mechanism of expiratory pressure generation during high-flow oxygen is dependent not only on gas flow rate, but also on the greater resistance offered by upper airways and patient’s expiratory flow. In tracheostomized patients, resistance is limited, and the generated pressure is minimal. Patient’s expiratory flow has wide inter-individual variability according to the resistive and elastic properties of the respiratory system and to the eventual recruitment of expiratory muscles [38]: thus, the pressure produced by HFOT_NASAL_ is variable among subjects, also if respiratory rate with HFOT_TRACHEAL_ is similar (Fig. 3) [39].

### Clinical consequences

Our study shows that the effects of HFOT_TRACHEAL_ are milder than HFOT_NASAL_, likely because the dedicated interface is completely open. HFOT_TRACHEAL_ allows to limit the negative swing in inspiratory airway pressure, but both the dead space washout and the generation of positive expiratory pressure are limited. From a clinical perspective, our findings suggest that a minimum gas flow of 50 L/min should be set during HFOT_TRACHEAL_ to slightly improve oxygenation and reduce respiratory rate, as compared to standard oxygen. Whether these mild physiologic effects are cost-effective and may clinically benefit the management of tracheostomized patients cannot be established from our data and should be addressed in further investigations.

### Limitations

First, we did not measure effectively delivered FiO_2_, as performed elsewhere [3]. As a result, the calculation of PaO_2_/FiO_2_ ratio may be subject to errors, especially if lower flows are used [40]. Nevertheless, our approach is clinically reproducible and we used a formula that has recently been shown to provide satisfactory correlation with actual FiO_2_ [24].

Second, we did not measure work of breathing by esophageal manometry [41]. However, esophageal catheter insertion in awake and spontaneously breathing patients may be challenging and eventually require some sedation. Importantly, during HFOT_NASAL_, changes in respiratory rate have been shown to reflect variations of the work of breathing [13, 33].

Third, there was no wash-out period between the applied interventions during HFOT_TRACHEAL_. However, our approach is consistent with previous investigations on the topic [18], and the randomized order of the interventions should have mitigated any carry-over effect on the observed results. Accordingly, the main outcomes of the study were not affected by the sequence of applied flow settings.

Fourth, during HFOT_NASAL_, absence of major leaks through the stoma was assessed by hand. Unfortunately, we had no other way to assess if minimal leaks were present. We believe, however, that even minimal leaks, if present, should not have affected tracheal pressure measurement. In fact, the tracheal pressure values we report are similar to nasopharyngeal pressure values measured in non-tracheostomized patients by others [13–15].

Finally, we showed that expiratory pressure increase due to HFOT_NASAL_ has wide inter-individual variability. Whether and to what extent expiratory flow limitation and expiratory muscles recruitment contribute to this is unknown and remains to be established in further investigations [38, 42].

### Conclusions

HFOT_TRACHEAL_ generates small flow-dependent improvement in oxygenation and increases in tracheal expiratory pressure. When compared to standard oxygen, a minimum flow of 50 L/min is needed during HFOT_TRACHEAL_ to improve oxygenation, increase expiratory pressure, limit inspiratory airway pressure swings and reduce respiratory rate. At same gas flow, HFOT_NASAL_ produces higher expiratory pressure than HFOT_TRACHEAL_.

## Data Availability

The datasets used and/or analyzed during the current study are available from the corresponding author on reasonable request.
